# The complete mitochondrial genome of *Allonychiurus kimi* (Lee, 1973) (Collembola: Onychiuridae)

**DOI:** 10.1080/23802359.2020.1846474

**Published:** 2021-01-12

**Authors:** Yun-Sik Lee, Taekjun Lee, Philjae Kim, Jino Son, June Wee, Yongeun Kim, Jinsol Hong, Minyoung Lee, Kijong Cho

**Affiliations:** a O-Jeong Eco-Resilience Institute, Korea University, Seoul, Republic of Korea; b Marine Biological Resource Institute, Sahmyook University, Seoul, Republic of Korea; c Division of Ecological Conservation, Bureau of Ecological Research, National Institute of Ecology, Choongnam, Republic of Korea; d Division of Environmental Science and Ecological Engineering, Korea University, Seoul, Republic of Korea

**Keywords:** Springtail, gene order, phylogeny, mitogenome

## Abstract

The complete mitochondrial genome of *Allonychiurus kimi* (Lee, 1973) was sequenced, assembled, and annotated. The mitochondrial genome of *A. kimi* is 14,386 bp in length and contains 13 protein-coding, 22 transfer RNA, and 2 ribosomal RNA genes*. A. kimi* was closely clustered with the following species of the family Onychiuridae: *Onychiurus orientalis*, *Orthonychiurus forlsomi*, and *Tetrodontophora bielanensis.*

Collembola, which has been reported to comprise about 9000 species worldwide (Bellinger 1996–2020), is the most abundant living organism in the soil ecosystem and plays an important role in soil formation and nutrient cycling (Rusek 1998; Filser 2002). The family Onychiuridae is composed of mostly soil-dwelling species that typically lack pigment, eyes, and a jumping organ (Hopkin 1997). Due to the limited number of mitochondrial genome records of Onychiuridae, the phylogenetic relationships of this family have not yet been resolved (Yao et al. 2020). In the present study, the mitochondrial genome of *Allonychiurus kimi* (Lee 1973) was sequenced, assembled, and annotated, and its molecular characteristics were described.

Specimens of *A. kimi* were first collected from a rice field in Ichon, Republic of Korea (32.267°N, 127.433°E) on 15 September 1996. Since then, *A. kimi* was cultured in the laboratory for approximately 24 years, and DNA from the samples used in the present study was deposited at Korea University, Seoul, Korea (specimen accession number KUEMCOL001). Mitochondrial DNA was extracted using the Qproteome Mitochondria Isolation Kit (Qiagen, Hilden, Germany) according to the manufacturer’s instructions and isolated using a QIAamp DNA Mini Kit (Qiagen). Mitochondrial DNA was amplified using the REPLI-g Mitochondrial DNA Kit (Qiagen). Next-generation sequencing (NGS) analysis was performed with genome analysis units at the National Instrumentation Center for Environmental Management of Seoul National University in Korea. A genomic library was constructed from the genomic DNA using a Kapa Hyper Prep Kit (Kapa Biosystems, Woburn, MA, USA) with paired-end reading followed by NGS using the Illumina Hi-Seq 2500 platform (San Diego, CA, USA). Phylogenetic analysis of the mitogenome nucleotide sequence dataset was performed using the maximum-likelihood (ML) method with RAxML 8.2 (Stamatakis 2014).

The mitogenome of *A. kimi* was 14,386 bp in length and contained 13 protein-coding genes (PCGs), 22 transfer RNA genes, and 2 ribosomal RNA genes. The overall nucleotide composition was 34.7% A, 16.9% C, 9.8% G, and 38.6% T, indicating an obvious A + T bias (73.3%). Among the 13 PCGs, four start codons were found: ATA (ATP6, ND4L, ND5, ND6), ATC (ATP8), ATG (COX3, ND4, CYTB), and ATT (COX1, COX2, ND1, ND2, ND3). Nine PCGs had a “TAA” stop codon; however, COX2, COX3, ND4L, and ND5 did not. The gene arrangement of *A. kimi* was identical to that of three other species of family Onychiuridae present in the NCBI database: *Onychiurus orientalis*, *Orthonychiurus forlsomi*, and *Tetrodontophora bielanensis*. The ML phylogenetic tree showed that a clade of the order Poduromorpha, including *A. kimi,* distinctly established a monophyletic clade (Figure 1). Among them, *A. kimi* was closely clustered with Onychiuridae species (Figure 1).

**Figure 1.**
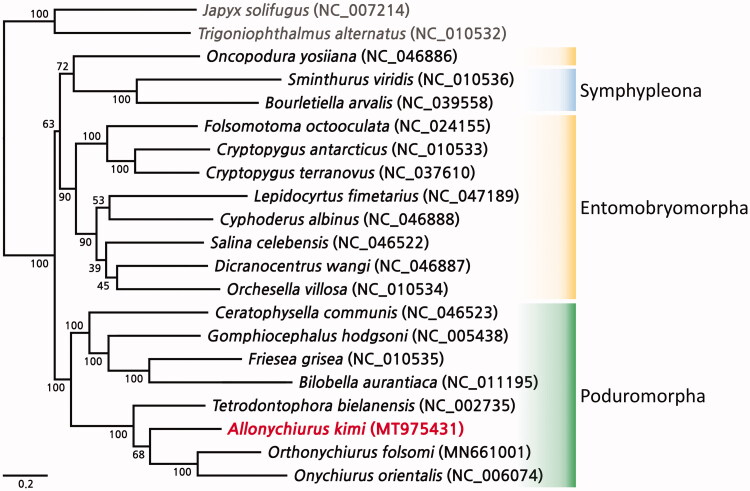
Phylogenetic analysis of *Allonychiurus kimi* and an additional 18 springtails performed using the maximum-likelihood method based on the nucleotide sequences of 13 protein-coding gene sequences. The bootstrap support values are indicated on each node.

## Data Availability

The data that support the findings of this study are openly available in “NCBI” at https://www.ncbi.nlm.nih.gov/, reference number MT975431.
